# Robotic-assisted sleeve gastrectomy with simultaneous Roux-en-Y cystojejunostomy in a patient with sever obesity and a pancreatic pseudocyst: a case report

**DOI:** 10.3389/fsurg.2023.1323704

**Published:** 2024-01-04

**Authors:** Zheng Zhang, Lun Wang, Zhiqiang Wei, Changyong E, Tao Jiang

**Affiliations:** Department of Hepatobiliary and Pancreatic Surgery, China-Japan Union Hospital, Jilin University, Changchun, China

**Keywords:** obesity, pancreatic pseudocyst, robotic surgery, sleeve gastrectomy, diabete

## Abstract

**Introduction:**

We tried to apply a new surgical method to treat obesity combined with pancreatic pseudocyst and achieved satisfactory results.

**Case and presentation:**

We report a case of a severely obese patient with pancreatic pseudocyst who underwent robotic-assisted sleeve gastrectomy, while the pseudocyst was incised and cyst-jejunostomy was performed. The operation was successful, and the patient was discharged on the 8th day after the procedure. There were no complications during the perioperative period. After 12 months of follow-up examinations, the patient's pancreatic pseudocyst disappeared. Additionally, there was a significant decrease in body weight, body mass index, and other indicators. As a result, obesity and related metabolic diseases were completely relieved.

**Conclusions:**

This case summarizes and presents the experience of using robotic bariatric surgery for the treatment of pancreatic pseudocyst. This case report indicates that this surgical procedure is both safe and effective for patients with pancreatic pseudocyst who also have obesity and related metabolic diseases.

## Background

Obesity is a risk factor for many metabolic diseases (1), including hypertension, diabetes, coronary heart disease, hyperlipidemia and so on. Acute pancreatitis (AP) is one of the most common digestive system diseases that require acute hospitalization in the world (2). The incidence rate is relatively high and is increasing year by year. The causes of the disease include gallstones, alcohol, hyperlipidemia, hypercalcemia, etc. Among them, hyperlipidemia is a common cause of the disease, and statistics have found that the incidence of hyperlipidemia pancreatitis (HLAP) accounts for all AP 6.17% (3). Therefore, obesity can be seen as one of the risk factors for hyperlipidemic acute pancreatitis.

Pancreatic pseudocyst (PP) refers to a cystic lesion formed after severe pancreatitis causes pancreatic juice to accumulate around the pancreas and is surrounded by proliferating fibrogranulation tissue (4). Surgery is the traditional method for the treatment of pancreatic pseudocyst. Because of its wide indications, thorough drainage, and the ability to biopsy to determine the nature of the lesion, it still plays an important role in the treatment of pancreatic pseudocyst (5). At present, bariatric surgery has been used as one of the treatment methods for recurrent obese acute pancreatitis, and sleeve gastrectomy as the most common bariatric surgical procedure and its options to convert to other bariatric surgical procedures (6).

Here, we report a case of a severely obese patient with pancreatic pseudocyst who underwent robotic-assisted sleeve gastrectomy, while the pseudocyst was incised and cyst-jejunostomy was performed. This case is reported to summarize and put forward the experience of robotic bariatric surgery for pancreatic pseudocyst.

## Case presentation

**Figure 1:**
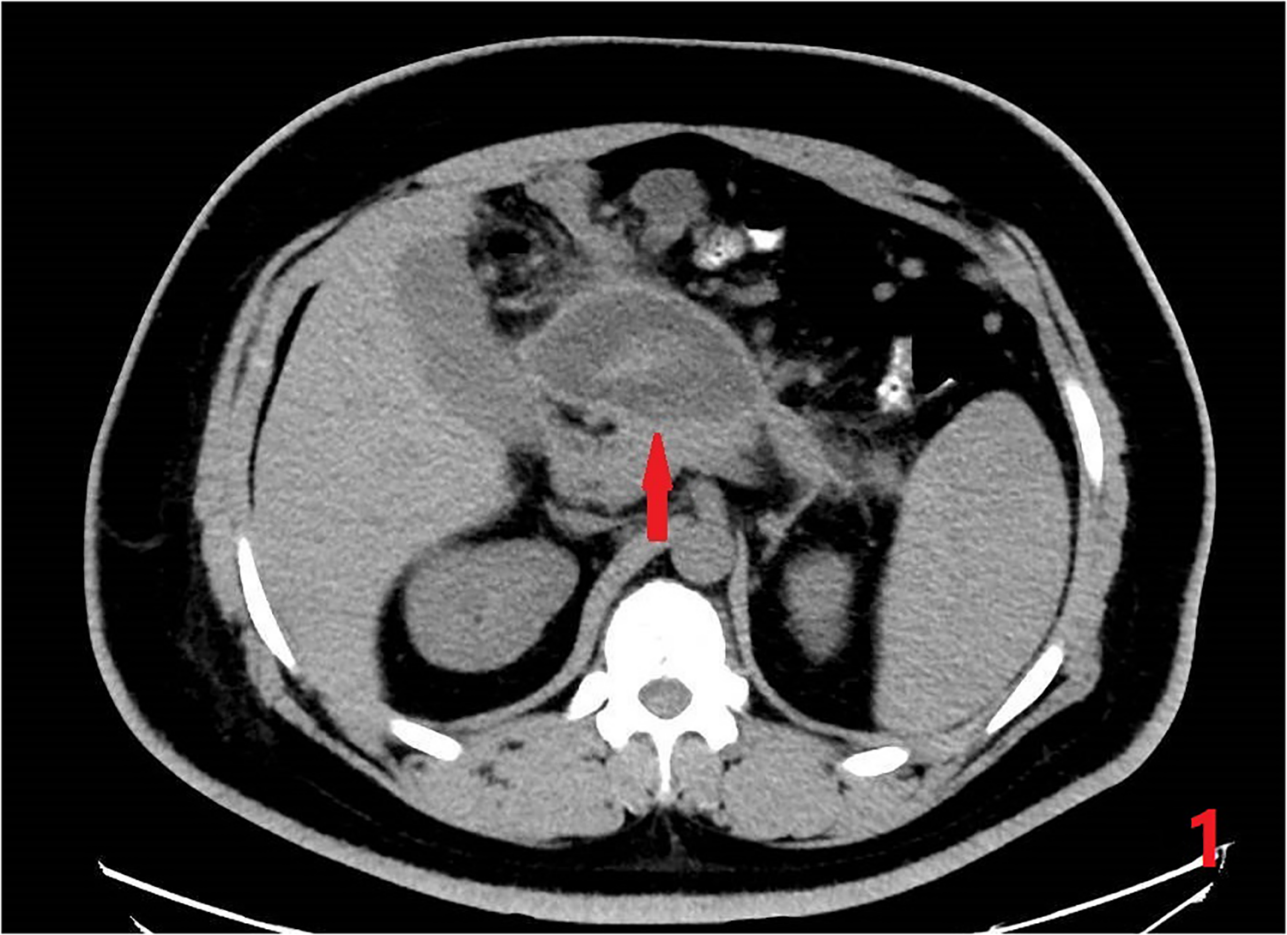
Preoperative CT. Pancreatic pseudocyst measuring 9.8*7.6*9.0 cm.

A 27-year-old man was admitted to the China-Japan Union Hospital of Jilin University with abdominal pain for 3 days. Basic physical examination was performed after admission: Height 174 cm, Weight 117 kg, Waist circumference 100 cm, Body mass index (BMI) 38.6 kg/m^2^. Tenderness under the xiphoid process in the upper abdomen, accompanied by mild rebound pain, without muscle tension. He had been hospitalized for acute pancreatitis 4 years ago, and the results of abdominal CT examination after admission showed: a cystic mass on the right side of the middle and upper abdomen, considering the possibility of pancreatic pseudocyst; fatty liver (Figure 1). Blood, urine amylase and white blood cells were significantly elevated, it is worth noting that his blood lipids also abnormally elevated. At the same time, a gallbladder ultrasound was performed to rule out biliary pancreatitis. Based on these basic conditions, he was diagnosed with acute pancreatitis, pancreatic pseudocyst, fatty liver, severe obesity, hypertriglyceridemia, type 2 diabetes, and hyperuricemia.

After symptomatic and supportive treatment, the patient's abdominal pain relieved, and blood and urine amylase returned to normal. In order to solve his pancreatic pseudocyst and related metabolic diseases, after multidisciplinary consultation and taking into account the degree of intra-abdominal adhesions of the patient, it was decided to perform robotic-assisted sleeve gastrectomy with simultaneous Roux-en-Y cystojejunostomy.

**Figure 2:**
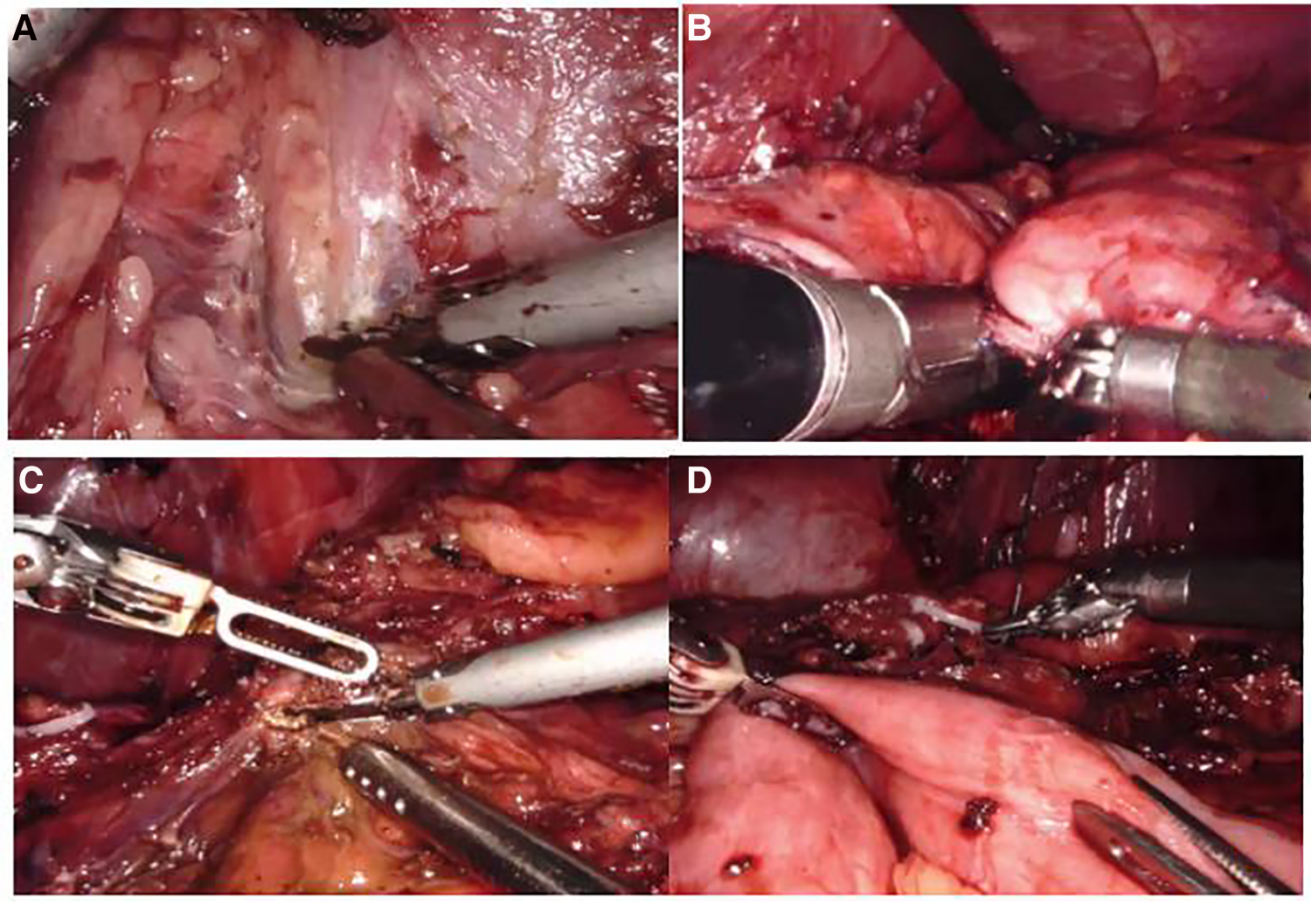
Intraoperative images and procedures: (**A**) pseudocyst was closely adhered to the posterior gastric wall. (**B**) Sleeve gastrectomy was performed. (**C**,**D**) Ultrasonic scalpel was used to incise the pseudocyst, and at the same time, the jejunum was lifted and anastomosed with the cyst.

After the anesthesia took effect, the abdominal cavity was first explored. A small amount of saponification plaques could be seen in the omentum. The ultrasonic scalpel dissected the gastrocolic ligament and exposed the pancreas. It was found that the posterior wall of the stomach was strongly adhered to the pancreas (Figure 2A). After careful separation of the adhesions, the greater curvature of the stomach and pancreatic pseudocyst were exposed, and a gastric sleeve resection was performed under the guidance of a 32 Fr bougie tube (Figure 2B). The jejunum was lifted up 40 cm away from the Treitz ligament, and Broun anastomosis was performed with the pseudocyst (Figures 2C,D), and the operation was completed. The operation time was 376 min and the amount of bleeding during surgery was 50 ml.

The patient engaged in activities on the 2nd day after the operation. An upper gastrointestinal radiography on the 4th day showed no abnormalities, allowing for the patient to begin consuming liquid foods. The patient was discharged smoothly on the 8th day after the operation.

**Figure 3:**
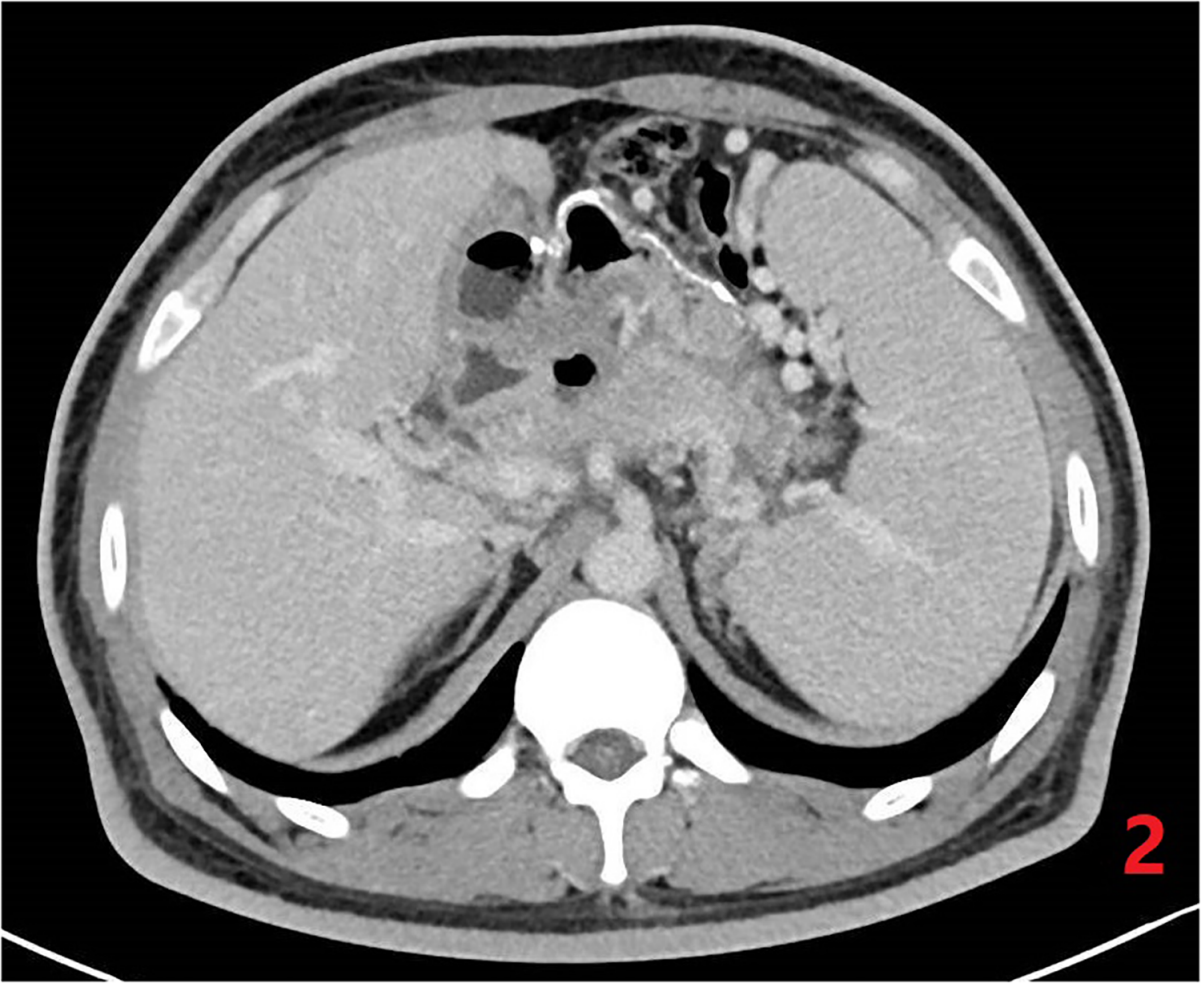
Postoperative CT of abdomen. Resolution of pseudocyst, but with regional portal hypertension.

The postoperative follow-up was 12 months. In this case, the Weight, BMI, Excess weight loss (%EWL) and Total weight loss (%TWL) decreased significantly at 3, 6 and 12 months after surgery, and high triglycerides Lipidemia, insulin resistance, and type 2 diabetes were all completely relieved, and the vitamin B 12 level returned to normal after operation, as shown in Table 1. No serious complications such as anastomotic leakage, bleeding, anastomotic obstruction and deep vein thrombosis occurred in this operation. The patient developed cholestasis 6 months after the operation. He was required to take ursodeoxycholic acid orally for choleretic treatment strictly according to the doctor's advice. After 12 months post-operation, ultrasound examination revealed no signs of cholestasis. Bone densitometry indicated normal bone densities in the lumbar spine and hip joints. However, an abdominal CT scan of the patient showed splenomegaly and multiple varicose veins in the fundus of the stomach, around the hilum of the spleen, and in the upper abdomen, as shown in Figure 3.

**Table 1:** The effect of weight loss and the remission of related metabolic diseases at 3, 6, and 12 months after operation.

	Preoperation	3 months	6 months	12 months
Weight (kg)	117	88	80	79
BMI (kg/m^2^)^a^	38.6	29.1	26.4	26.1
EWL (%)^a^	–	61.1	78.1	80.2
TWL (%)^a^	–	24.8	31.6	32.5
FPG (mmol/L)^a^	12.95	5.18	5.63	5.02
HbA1c (%)^a^	8.60	5.50	5.20	–
Uric acid (μmol/L)	587.25	595.02	498.73	509.79
Triglyceride (mmol/L)	8.14	2.19	1.52	1.24
Cholesterol (mmol/L)	9.95	4.61	4.39	3.84
Vitamin B12 (pmol/L)	425.9	591.6	667.0	705.5

^a^ BMI, body mass index; EWL, excess weight loss; TWL, total weight loss; FPG, fasting plasma glucose; HbA1c, glycosylated hemoglobin.

## Discussion

Patients diagnosed with HLAP exhibit a younger onset age and a higher prevalence of male patients. Additionally, HLAP is often associated with metabolic diseases such as obesity, diabetes, and fatty liver. These correlations may be attributed to the poor lifestyle habits of this particular age group, including irregular sleeping patterns, high stress levels, and a diet high in fat. Hyperlipidemia is not only a risk factor for AP, but also for its recurrence. Poor control of blood lipids and blood sugar may be related to relapse (7). Therefore, the treatment of HLAP should focus not only on the acute phase but also on the rehabilitation phase, with the aim of better controlling blood lipids, blood glucose, and other related metabolic indicators (8).

PP are frequently encountered cystic lesions of the pancreas that occur as a result of AP. In most cases, PP can be managed conservatively and resolve spontaneously without the need for surgical intervention. However, if the size of the cyst exceeds 6 cm and does not resolve within 6 weeks, surgical treatment should be considered (9). The current surgical treatment options for cyst drainage include endoscopic drainage, traditional external drainage, and internal drainage. Of these options, Roux-en-Y Cystojejunostomy is the most commonly used and considered the most optimal surgical method (10, 11).

The case in this article is that the blood lipids are not well controlled, and AP recurs, which eventually leads to the formation of PP. Therefore, for him, the treatment of metabolic diseases is equally important. Bariatric metabolic surgery appeared in the 1950s and has become an effective means of treating severe obesity and its related metabolic diseases after continuous improvement by bariatric surgeons around the world (12). Among many weight loss surgeries, SG has been rapidly popularized due to its simple operation, less damage to physiological structures, high safety, and obvious curative effect, and has become the most commonly used bariatric metabolic surgery method, accounting for about 50% (13).

We have also considered a “two-stage” approach to solving this problem, which involves performing sleeve gastrectomy first and then performing endoscopic drainage after a period of time. This approach appears to be safe and feasible. However, we had to abandon this plan because the patient was unwilling to undergo a second surgery. So we chose to use Sleeve Gastrectomy with Simultaneous Roux-en-Y Cystojejunostomy to solve his problem.

Almerie et al. (14) reported a case of Laparoscopic Sleeve Gastrectomy with Simultaneous Laparoscopic Cystogastrostomy. The original plan for the patient was to undergo a laparoscopic Roux-en-Y gastric bypass surgery and drain the pseudocyst into the remnant stomach, but during the operation, adhesions were discovered between the pancreatic pseudocyst and the stomach, leading the medical team to switch to sleeve gastrectomy. The patient was discharged four days after the surgery. The patient experienced gastrointestinal bleeding, which was initially treated conservatively. However, when this failed, a CT angiography was performed, and splenic aneurysm bleeding was suspected. The patient then underwent vascular interventional embolization and was discharged a few days later without complications. After three months of follow-up, the patient had a successful recovery. Similarly, we also consider that the special anatomical relationship between the stomach and pancreas and the severity of intra-abdominal inflammatory adhesions may increase the difficulty and risk of traditional laparoscopic surgery. At this time, the da Vinci robotic surgery system, as the latest achievement of minimally invasive surgery, has entered our field of vision with its unique advantages.

The magnified view, improved ergonomics and dexterity offered by robotic platforms might facilitate the uptake of minimally invasive procedures (15). The robotic surgery system makes up for the technical difficulties of traditional laparoscopic pancreatic surgery and bariatric surgery to a certain extent (16, 17). Robotic surgery systems are often used in pancreatic tumor surgery, but studies have also proved (18) that they are also effective and feasible for surgical treatment of pancreatic pseudocysts. Robotic surgery involves transforming the tactile feedback of open surgery into visual feedback, which helps to prevent excessive stretching of tissues and promotes fine dissection and cutting. This technique is beneficial in minimizing tissue damage and improving surgical precision. Therefore, this patient benefited significantly from the robotic surgery. He left the bed on the 2nd day after the operation. On the 4th day, he underwent upper gastrointestinal angiography without abnormalities, and then he took a liquid diet. He was discharged from the hospital on the 8th day after the operation. There were no related complications during the perioperative period occur.

After one year of follow-up, the patient's EWL% and TWL% were 80.2% and 32.5%, respectively, and the patient's hyperlipidemia, type 2 diabetes and other related metabolic diseases were completely relieved. However, the abdominal CT examination of the patient one year after the operation showed regional portal hypertension and splenomegaly. This condition may be attributed to chronic inflammation of the pancreas over a long period of time, along with the presence of fibrotic material surrounding the main portal vein, splenic vein, and superior mesenteric vein. Consequently, this can result in pancreatic portal hypertension.

## Summary and conclusion

In conclusion, the surgical method outlined in this article is safe, feasible, and effective. The Da Vinci robotic surgery system played a unique role in the treatment of this patient, and the postoperative long-term effect was good. Worthy of support, but signage must be strictly followed and operating specifications. In addition, this case also promoted the integration of obesity metabolic surgery and pancreatic surgery and other related fields, and provided a new idea for the development of obesity metabolic surgery.

## Data Availability

The original contributions presented in the study are included in the article/Supplementary Material, further inquiries can be directed to the corresponding author.
